# Nutraceutical benefits and neuro-protective potent of four colored peppers (*Capsicum annuum var. grossum*)

**DOI:** 10.37796/2211-8039.1654

**Published:** 2025-06-01

**Authors:** Ya-Chen Yang, Chih-Lung Lin, Mei-Chin Yin

**Affiliations:** a Department of Food Nutrition and Health Biotechnology, Asia University, Taichung, Taiwan; b Department of Neurosurgery, Asia University Hospital, Taichung, Taiwan; c Office of Research and Development, Asia University, Taichung, Taiwan; d Department of Medical Research, China Medical University Hospital, China Medical University, Taichung, Taiwan

**Keywords:** Colored peppers, Phytochemicals, Vitamin C, Nutraceutical benefits, Neuro-protection

## Abstract

**Background:**

Four colored peppers (*Capsicum annuum var. grossum*), orange, purple, yellow and red, are plant foods served as salad or stir-fry for meals in Taiwan and many countries.

**Purpose:**

This study aimed to investigate the multiple nutraceutical properties of aqueous extracts prepared from colored peppers.

**Methods:**

Vitamin C content and phytochemical profiles of these peppers were analyzed. *In vitro* effects of anti-oxidative, anti-α-amylase, anti-α-glucosidase, anti-lipase and anti-acetylcholinesterase (AchE) of pepper aqueous extracts at 0.25, 0.5 and 1 mg were evaluated. The neuronal protective potent of pepper aqueous extracts at 0.5 and 1 mg in high glucose treated nerve growth factor (NGF)-differentiated PC12 cells were examined.

**Results:**

Vitamin C content in these peppers was in the range of 60–96 mg/100 g fresh weight. The content of phenolic acids, flavonoids, anthocyanins and triterpenoids in these peppers was in the range of 860–2185 mg/100 g dry weight. Pepper aqueous extracts at 0.25, 0.5 and 1 mg exhibited concentration-dependent radical scavenging effects, ironchelating effects and reducing power, as well as effectively inhibited α-amylase, α-glucosidase, lipase and AchE activities. High glucose increased Bax mRNA expression, decreased mitochondrial membrane potential and Na^+^-K^+^ ATPase activity, caused DNA fragmentation and massive Ca^2+^ release, stimulated oxidative and inflammatory responses, and led to death of NGF-differentiated PC12 cells. Pre-treatments of pepper aqueous extracts at 0.5 and 1 mg reversed these changes, and increased the viability of NGF-differentiated PC12 cells.

**Conclusions:**

These novel findings suggest that colored peppers offered many bio-functions, which might benefit the prevention of diabetes associated complications such as diabetic neuropathy.

## 1. Introduction

**Fig. 1:**
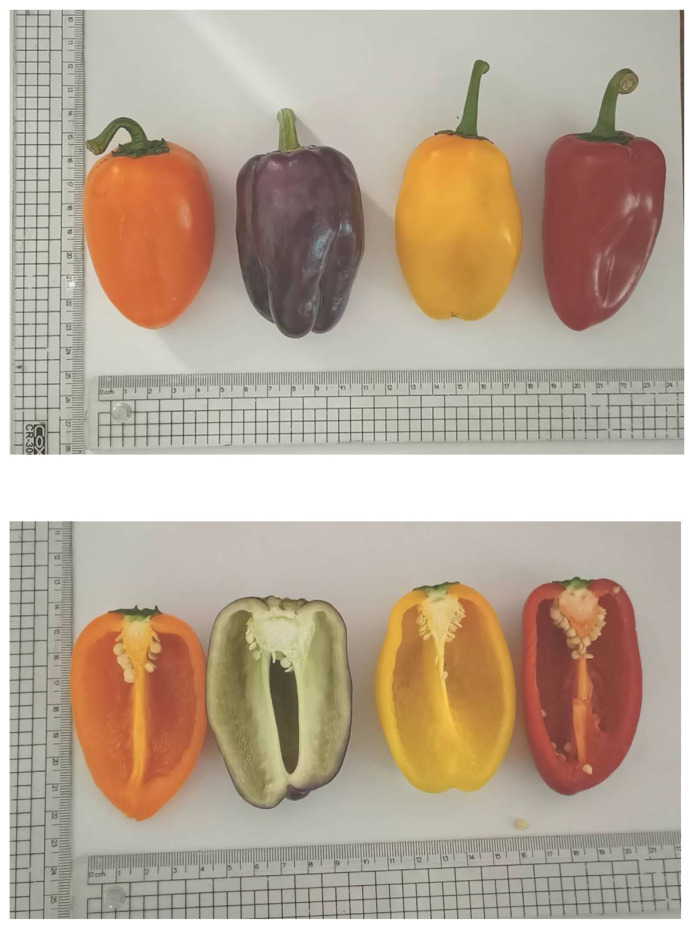
Pictures of four colored peppers, orange, purple, yellow and red.

Four edible colored peppers (*Capsicum annuum var. grossum*), as shown in Fig. 1, are served as salad or stir-fry for meals in Taiwan. So far, less information is available regarding their nutraceutical composition and effects. It is documented that phytochemicals presented in plant foods including vitamin C, phenolic acids, flavonoids, anthocyanins or triterpenoids enhance the nutritional and/or medical values of these plant foods via providing important bio-functions such as anti-oxidative, anti-diabetic, anti-inflammatory or even anti-cancer activities [1,2]. Thus, understanding phytochemical profiles of these colored peppers may contribute to their future applications.

*In vitro* anti-oxidative activities include scavenging free radicals, iron-chelating effects and reducing power [3]. If plant foods possess these activities, the intake of these foods may reinforce consumer’s defensive capability against oxidative damage [4]. α-Amylase and α-glucosidase are two key digestive enzymes responsible for glucose metabolism. Increased activity of these enzymes facilitates carbohydrate (CHO) degradation and glucose formation, which in turn disturbs glycemic control and favors diabetic progression [5]. The inhibition upon these enzymes has been considered as therapeutic targets for developing agent(s) against diabetes [6]. Pancreatic lipase converts dietary triglycerides to glycerol and fatty acids, which consequently promotes the uptake of triglycerides into circulation or organs. The limitation upon this enzyme is a target for preventing lipid metabolism disorders such as hyperlipidemia and obesity [7]. Acetylcholinesterase (AchE) is the key enzyme responsible for acetylcholine hydrolysis. The increased AchE activity decreases acetylcholine level in brain, which leads to the pathogenic development of Alzheimer’s disease (AD) [8]. Thus, if the aqueous extracts prepared from colored peppers could restrict the activity of these crucial enzymes, these peppers might be able to prevent and ameliorate diabetes, obesity and AD.

Diabetic neuropathy (DN), one of diabetic complications, is characterized by sensorimotor polyneuropathy such as chronic pain, nerve degeneration and sensory loss [9]. The molecular and cellular mechanisms responsible for DN progression included hyperglycemia-induced mitochondrial dysfunctions, oxidative stress and neuroinflammation [10,11]. Consequently, neurons in nerve tissues such as brain are malfunctions or even destroyed. Thus, any agent with the effects to stabilize bio-membranes, mitigate oxidative and inflammatory injury may potentially protect neurons and retard the progression of DN. Phytochemicals from plants offers abundant natural antioxidants, and their applications upon DN improvement has been attracted [12,13]. Therefore, nerve growth factor (NGF)-differentiated PC12 cells, a neuronal cell model [14], were used to evaluate the protective effects of pepper aqueous extracts against high glucose. The impact of pepper aqueous extracts upon cell survival, bio-membrane stability, mRNA expression of Bcl-2 and Bax, Ca^2+^ homeostasis and the alleviation of oxidative and inflammatory stress under high glucose condition was examined. Aqueous extract was used because it has the following advantages, easy preparation, without chemical residue and high safety. Furthermore, if these peppers possess anti-diabetic and neuroprotective potent, they might be able to provide nutritional and medicinal benefits for consumers.

## 2. Materials and methods

### 2.1. Materials

Fresh peppers were purchased from farms in Nantou County, Taiwan, in June–August 2019. Each pepper at 300 g was chopped, mixed with 500 mL distilled water and homogenized by a Waring blender at high speed. The homogenate was remained for 12 h at 25 °C, and filtrated through a sterile filter paper. The collected filtrate was further dried to fine powder through a frozen air-drying process. The powder, defined as aqueous extract, was used to determine phytochemical profiles, *in vitro* anti-oxidative and anti-enzymes activities, and NGF-differentiated PC12 cells experiments.

### 2.2. Measurement of vitamin C content

Vitamin C content was measured by an Association of Official Analytical Chemistry method [15]. Briefly, fresh pepper at 150 g was chopped and homogenized. The homogenate was further mixed with a 300 mL 2 % oxalic acid solution. After filtered through a Whatman filter paper, the filtrate was titrated with freshly prepared 2,6-dichloroindophenol at 0.1 % until the appearance of a pink color. Vitamin C content was calculated and expressed as mg/100 g fresh weight.

### 2.3. Determination of phytochemicals

Total phenolic acids and total flavonoids were assessed via a Folin-Ciocalteu method and a colorimetrical method, respectively, in which absorbance at 755 nm and 420 nm was read [16]. The levels of total phenolic acids and total flavonoids were expressed as mg gallic acid equivalents/100 g dry weight (DW), and mg quercetin equivalents/100 g DW, respectively. Total anthocyanins were quantified by a pH differential method, absorbances at 510 and 700 nm were read [17]. Result was calculated as cyanidin-3-glucoside equivalents/100 g DW. The vanillin/glacial acetic acid method was used to measure the content of total triterpenoids, and absorbance at 548 nm was recorded [18]. Result was reported as mg ursolic acid equivalents/100 g DW. Pepper aqueous extracts used for experiments were standardized by the content of total anthocyanins and total triterpenoids.

### 2.4. Assays for 2,2-Diphenyl-1-picrylhydrazyl (DPPH) radical scavenging effects, iron-chelating effects and reducing power

Pepper aqueous extracts at 0.25, 0.5 and 1 mg were mixed with distilled water at 500 μL, and this mixture was further reacted with DPPH ethanolic solution at 500 μL of 60 μM.

After a 30-min incubation at room temperature, the absorbance at 540 nm was detected by a microplate reader, and ascorbic acid (AA) was applied as a positive control. Data are expressed as AA equivalents (AAE)/g DW. The method of Le et al. [19] was used to determine iron-chelating effects. Pepper aqueous extracts at 0.25, 0.5 and 1 mg were added into a 2 mL solution consisted of 9 mM ferrous sulfate, 30 mM hexamine and 30 mM potassium chloride. After a 10-min incubation at room temperature, samples were reacted with 1 mM tetramethyl murexide at 200 μL. The absorbance at 485 nm was monitored. Ethyl-enediaminetetraacetic acid (EDTA) at 1 mg was used as a standard, and result was shown as percentage of EDTA. Reducing power was measured according to the method of Andrés et al. [20]. Pepper aqueous extracts at 0.25, 0.5 and 1 mg were added into a solution containing 1 % potassium ferricyanide at 1 mL and 200 mM phosphate buffered saline (PBS) at 1 mL, and followed by a 20-min incubation at 50 °C. After further mixed with 10 % trichloroacetic acid (TCA) at 1 mL, sample was centrifugated at 650 x g for 10 min. Supernatant was collected and mixed with a solution containing 0.1 % ferric chloride at 1 mL and deionized water at 1 mL. The absorbance at 700 nm was measured, which was directly reported as data.

### 2.5. Measurement of α-amylase, α-glucosidase, Lipase and AchE activities

The method of Akanji et al. [21] was used to detect the inhibitory effects upon α-amylase and α-glucosidase activities. In the assay for α-amylase inhibition, pepper aqueous extracts at 0.25, 0.5 and 1 mg were dissolved in 1.5 mL 20 mM PBS (pH 6.9). This mixture was reacted with α-amylase at 0.5 mg/mL. After a 30-min incubation at room temperature, 1 % soluble potato starch at 2 mL was added as a substrate for reaction. After a 10-min incubation at room temperature, 1 % dinitrosalicylic acid at 2 mL was added to stop the reaction. After a 5-min heating in boiling water bath, the developed orange-red color was recorded by monitoring the absorbance at 550 nm. In the assay for α-glucosidase inhibition, α-glucosidase replaced α-amylase, and the substrate was 3 mM p-nitrophenyl glucopyranoside at 2 mL. After a 20-min incubation at 37 °C, 2 mL Na_2_CO_3_ solution at 100 M was added to stop the reaction. The absorbance at 405 nm was recorded. The inhibitory effect (%) was determined according to the equation, (A_control_ − A_sample_)/A_control_ x 100. For both enzyme assays, controls were PBS buffer. The method of de Camargo et al. [22] was applied to assay the anti-lipase activity. p-Nitrophenyl butyrate (p-NPB) at 5 mM was prepared in dimethyl sulfoxide (DMSO). Type 2 porcine pancreatic lipase at 5 mg/mL was prepared in distilled water, and followed by a 5-min centrifugation at 10,000 x g. The supernatant was collected as an enzyme solution. Pepper aqueous extracts at 0.25, 0.5 and 1 mg were dissolved in 500 μL 10 % DMSO first, and mixed with 450 μL Tris–HCl buffer and 50 μL enzyme solution. After a 15-min incubation at 37 °C, 30 μL p-NPB was added. After a 25-min incubation at 37 °C, the absorbance at 405 nm was recorded. Orlistat (ORL) at 1 mg was used as the standard and the inhibitory effect was repressed as a percentage of ORL. An assay kit purchased from BioVision Co. (Milpitas, CA, USA) was used to measure the inhibitory effects of pepper aqueous extracts upon AchE activity. Pepper aqueous extracts at 0.25, 0.5 and 1 mg were added into a 2 mL PBS (20 mM, pH 6.9) first. Pepper sample at 100 μL was mixed with 100 μL of assay buffer. Donepezil hydrochloride (DH) at 1 mg was used as a standard. After a 20-min incubation at 37 °C, the absorbance at 570 nm was measured. Inhibitory percentage was calculated as follows: (A_control_ − A_sample_)/A_control_ × 100.

### 2.6. Cell line study

Pepper aqueous extracts at 0.25 mg did not exhibit remarkable effects in examined *in vitro* bio-activities. Thus, this concentration, 0.25 mg, was not used in cell line study. PC 12 cells obtained from American Type Culture Collection (Rockville, MD, USA) were cultured in Dulbecco’s modified Eagle’s medium (DMEM, Difco Lab., Detroit, MI, USA), containing fetal bovine serum at 5 %, heat-inactivated calf serum at 10 %, NaHCO_3_ at 1.5 g/L, penicillin at 100 units/mL, streptomycin at 100 units/mL and glucose at 5.5 mM. Cells were cultured under an air: CO2 (95:5) condition at 37 °C. NGF (99 %) at 50 ng/mL was reacted with cells at 10^5^/mL, and this mixture was further incubated for 5 d at 37 °C for differentiation. Differentiated cells were plated onto culture dishes for experiments. Pepper aqueous extracts at 0.5 and 1 mg was added into 50 mL DMEM for cell culture, and followed by 48 h incubation at 37 °C. Then, those cells were cultured with medium containing 33mM high glucose for 24 h at 37 °C. Normal groups were NGF-differentiated PC 12 cells treated with 5.5 mM glucose and without pepper aqueous extracts. Control groups were NGF-differentiated PC 12 cells treated with 33 mM glucose and without pepper aqueous extracts.

### 2.7. Determination of viability and lactate dehydrogenase (LDH) activity

Cells at 10^5^/mL was reacted with 3-(4,5-dimethylthiazol-2-yl)-2,5-diphenyltetrazolium bromide at 0.25 mg/mL for 3 h at 37 °C. The formazan product was quantified by reading the absorbance at 570 nm via a microplate reader. Cell viability of normal groups was considered as 100 %, and the viability of other groups was reported as a percentage of normal groups. Cells were homogenized, centrifugated and supernatants were collected. Protein content of cell supernatants was analyzed by a protein assay kit (Sigma–Aldrich Co., St. Louis, MO, USA).

LDH activity in supernatants was determined by an assay kit (Roche Diagnostics, Penzberg, Bavaria, Germany).

### 2.8. Determination of mRNA expression

mRNA expression of Bcl-2 and Bax was determined by real-time polymerase chain reaction (RT-PCR). Total RNA was isolated, and its concentration was determined by reading 260 nm absorbance. A synthesis kit (Takara Biomedical Technology Co., Ltd., Beijing, China) was used to synthesize cDNA by 5 μg RNA. Then, cDNA was further applied to process RT-PCR through activating 10 min at 95 °C, denaturing 15s at 95 °C, annealing and elongating 1 min at 56 °C. Bcl-2 and Bax were performed 40 cycles. Glyceraldehyde-3-phosphate dehydrogenase (GAPDH, a house keeping gene) was performed 32 cycles. Sense and antisense primers were as follow: GAPDH, forward, 5′-AGA GGC AGG GAT GTT CTG-3′, reverse, 5′-GAC TCA TGA CCA CAG TCC ATG C-3′; Bcl-2, forward, 5′-GTG GAT GAC TGA GTA CCT GAA C-3′, reverse, 5′-GAG ACA GCC AGG AGA AAT CAA-3′; and Bax, forward, 5′-GCT GAT GGC AAC TTC AAC TG-3′, reverse, 5′-ATC AGC TCG GGC ACT TTA G-3′. A real-time sequence detection system was applied to detect the generated fluorescence intensity.

### 2.9. Determination of mitochondrial membrane potential (MMP), Na^+^-K^+^ ATPase activity and DNA fragmentation

Cells at 10^5^/mL were mixed with Rhodamine 123 at 100 μg/L. After a 30-min incubation at 37 °C, the value of mean fluorescence intensity (MFI), an MMP indicator, was assessed via a Beckman Coulter flow cytometry (Fullerton, CA, USA). The activity of Na^+^-K^+^ ATPase was assayed by monitoring the release of inorganic phosphate from ATP. Cell pellet was collected after a 5000×g centrifugation at 4 °C for 10 min. Mitochondrial fraction was separated from pellet via a mitochondria isolation kit purchased from Sigma–Aldrich Co. (St. Louis, MO, USA). Then, mitochondria were added into a 30 mM Tris–HCl buffer (pH 7.4). Assay process was initiated by adding ATP, and followed by a 15-min incubation at 37 °C. Assay was terminated by adding 0.5 mL TCA at 10 %. The absorbance at 640 nm was read via a fluorophotometer. A cell death detection kit purchased from Roche Molecular Biochemicals (Mannheim, Germany) was used to measure DNA fragmentation. After a 30-min suspending in lysis buffer and a 10-min centrifugation at 250×g, 20 μL cell supernatant was reacted with 80 μL immunoreagents. After a 2-h incubation at room temperature, substrate was added to react with this mixture for 15 min. The absorbance at both 405 nm and 490 nm were recorded. DNA fragmentation was expressed as a fold of normal groups.

### 2.10. Measurement of Intracellular Ca^2^^+^ level

Intracellular Ca^2+^ level (nM) was determined by the method described in Yun and Jeong [23]. Cell homogenate was mixed with 1 % bovine serum albumin, 0.1 % DMSO and 2 mg Fura-2-acetoxymethyl ester, and followed by a 60-min incubation under dark condition at 37 °C. The fluorescence value was measured at 510 nm as emission wavelength, and at 340 nm and 380 nm as excitation wavelengths. The ratio of fluorescence values, F_340_/F_380_, was applied as an indicator of intracellular Ca^2+^ level. F_340_ and F_380_ were the fluorescence values of Ca^2+^-bound form and Ca^2+^-free form, respectively.

### 2.11. Measurements of oxidative and inflammatory factors

Cell supernatants were collected after centrifugation. Glutathione (GSH), tumor necrosis factor (TNF)-alpha, interleukin (IL)-1beta and IL-6 levels, glutathione peroxidase (GPX) and glutathione reductase (GR) activities in supernatants were measured by assay kits obtained from Wako Chem. Co. (Tokyo, Japan). Level of reactive oxygen species (ROS) was measured by mixing cell homogenate at 100 μL with 2′,7′-dichlorofluorescein diacetate at 2 mg/mL, and incubating at 37 °C for 30 min. The fluorescence values at 488 and 525 nm were monitored by a microplate reader. The relative fluorescence unit (RFU) per mg protein was directly reported.

### 2.12. Statistical analyses

Each value was obtained from seven different preparations (n = 7). Data were reported as means ± standard deviation (SD). Statistical analyses were handled by using SAS (SAS Institute, Cary, NC, USA) and one-way analysis of variance (ANOVA). Least Significance Difference Test was performed to determine the differences among means.

It was considered as significant when *P* < 0.05.

## 3. Results

### 3.1. Vitamin C content and phytochemical profiles

As shown in Table 1, vitamin C content in four peppers was in the range of 60–96 mg/100 g fresh weight, and red pepper had greater vitamin C content than other peppers (*P* < 0.05). The content of total phenolic acids, total flavonoids, total anthocyanins and total triterpenoids was in the range of 919–1081, 903–1416, 1453–1960 and 1375–2185 mg/100 g DW. Purple pepper had greater total anthocyanins and total triterpenoids levels than other peppers (*P* < 0.05).

### 3.2. In vitro anti-oxidative effects

**Fig. 2:**
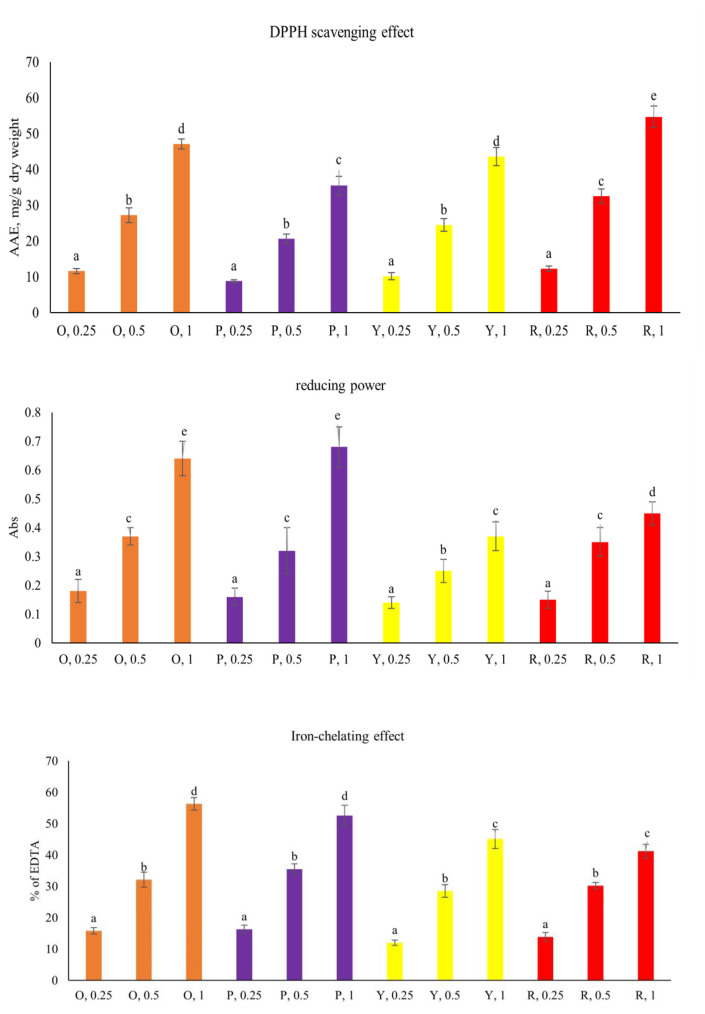
In vitro anti-oxidative activities of 0.25, 0.5 and 1 mg aqueous extracts prepared from orange (O), purple (P), yellow (Y) and red (R) peppers. Data were expressed as 3 mean ± SD (n = 7). ^a–e^Values among bars without a common letter differ, P < 0.05.

As shown in Fig. 2, each pepper concentration-dependently increased its DPPH scavenging effects, iron-chelating effects and reducing power (*P* < 0.05). Red pepper had greater DPPH scavenging effects than other peppers at 1 mg treatments (*P* < 0.05). Orange and purple peppers had greater iron-chelating effects and reducing power than red and yellow peppers at 1 mg treatments (*P* < 0.05).

### 3.3. Inhibitory Effects upon α-amylase, α-glucosidase, Lipase and AchE activities

**Fig. 3:**
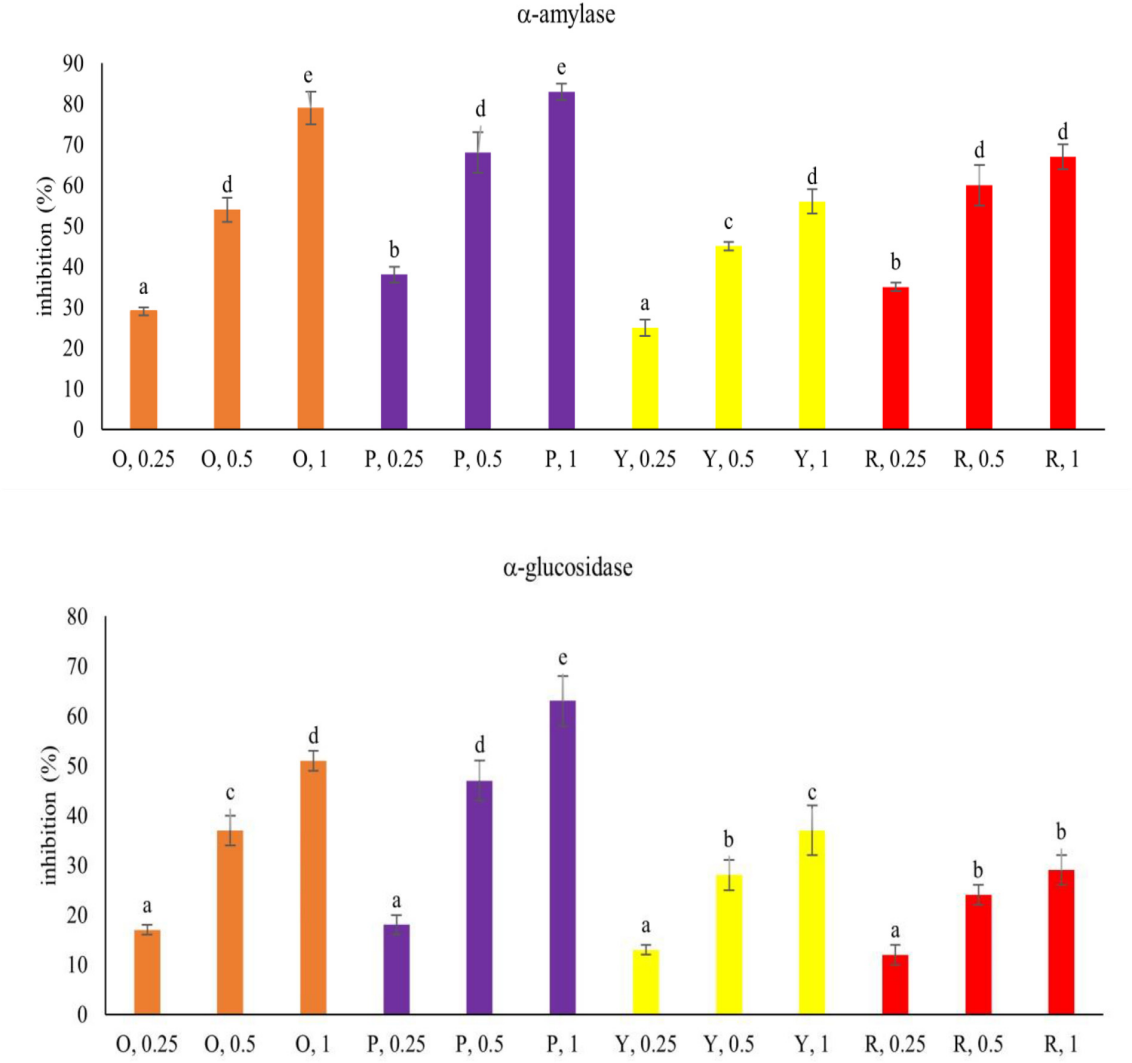

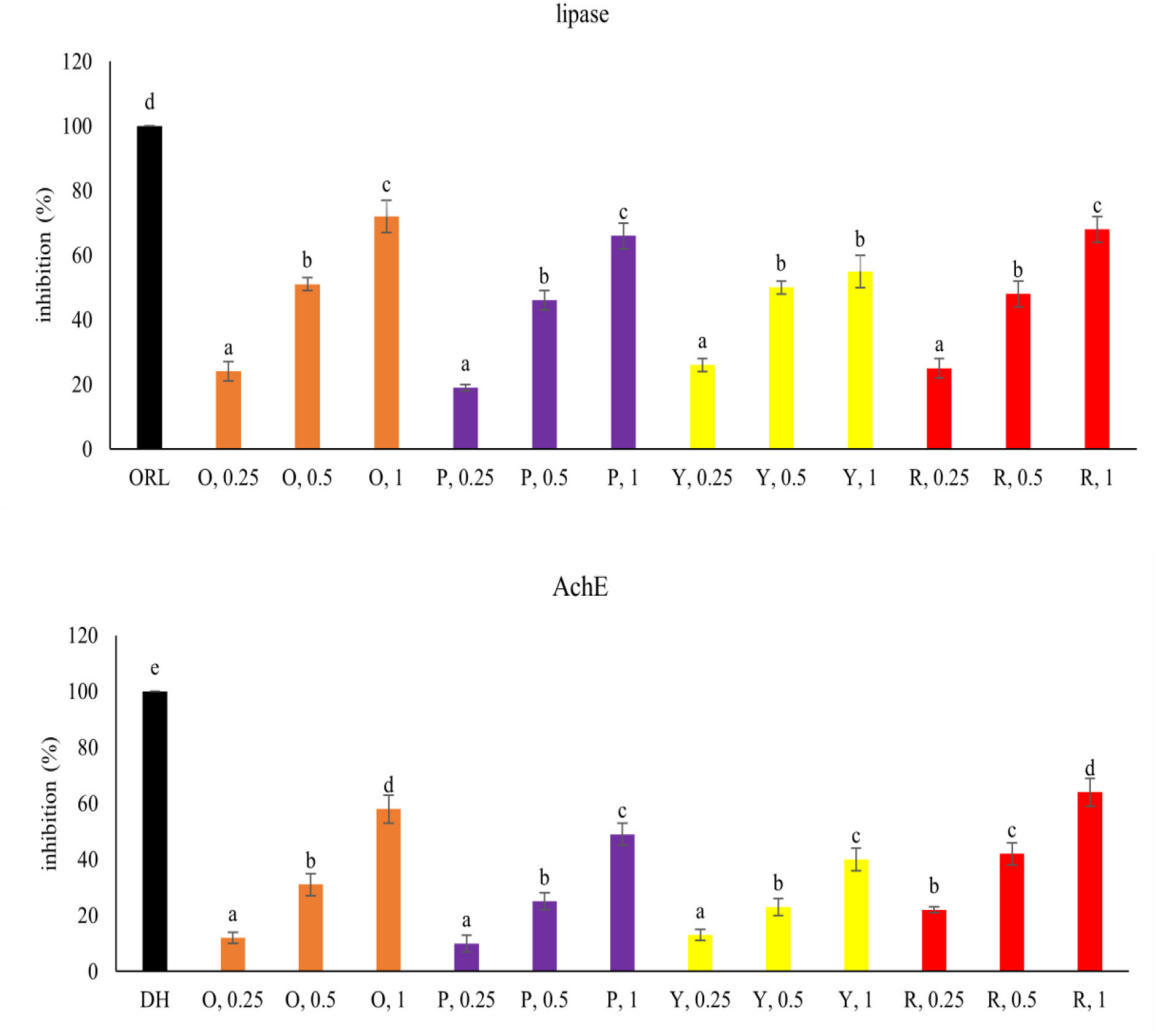
Inhibitory effects of 0.25, 0.5 and 1 mg aqueous extracts prepared from orange (O), purple (P), yellow (Y) and red (R) peppers upon the activity of α-amylase, α-glucosidase, lipase and AchE. Orlistat (ORL) at 1 mg was applied for anti-lipase comparison. Donepezil hydrochloride (DH) at 1 mg was applied for anti-AchE comparison. Data were expressed as mean ± SD (n = 7). ^a–e^Values among bars without a common letter differ, P < 0.05.

Each pepper at test concentrations exhibited inhibition upon α-amylase, α-glucosidase, lipase and AchE activities (Fig. 3, *P* < 0.05). Purple pepper at 1 mg had greater inhibitory effects upon the activity of α-amylase and α-glucosidase than other peppers at the same concentration (*P* < 0.05). Orange, purple and red peppers at test concentrations displayed similar inhibitory effects against lipase (*P* > 0.05). Orange and red peppers at 1 mg had greater anti-AchE activities than other two peppers (*P* < 0.05).

### 3.4. Viability of NGF-differentiated PC12 Cells

**Fig. 4:**
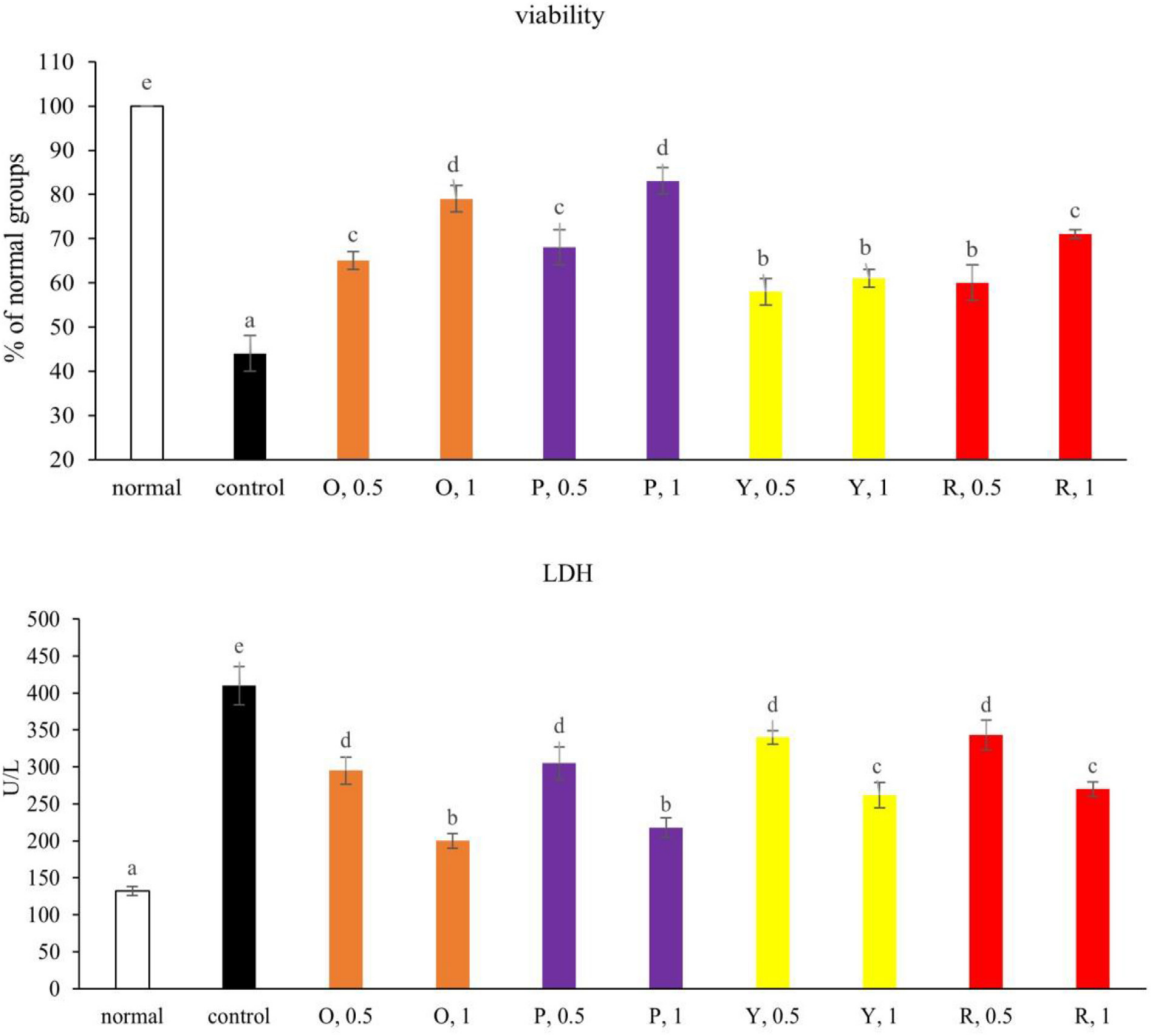
Effects of aqueous extracts prepared from orange (O), purple (P), yellow (Y) and red (R) peppers upon cell viability and LDH activity. NGF-differentiated PC12 cells were pre-treated with aqueous extracts at 0.5 and 1 mg for 48 h at 37 °C, and followed by 33 mM glucose treatment for 24 h. Normal groups were NGF-differentiated PC12 cells treated with 5.5 mM glucose and without aqueous extracts. Control groups were NGF-differentiated PC12 cells treated with 33 mM glucose and without aqueous extracts. Data were expressed as mean ± SD (n = 7). ^a–e^Values among bars without a common letter differ, P < 0.05.

**Fig. 5:**
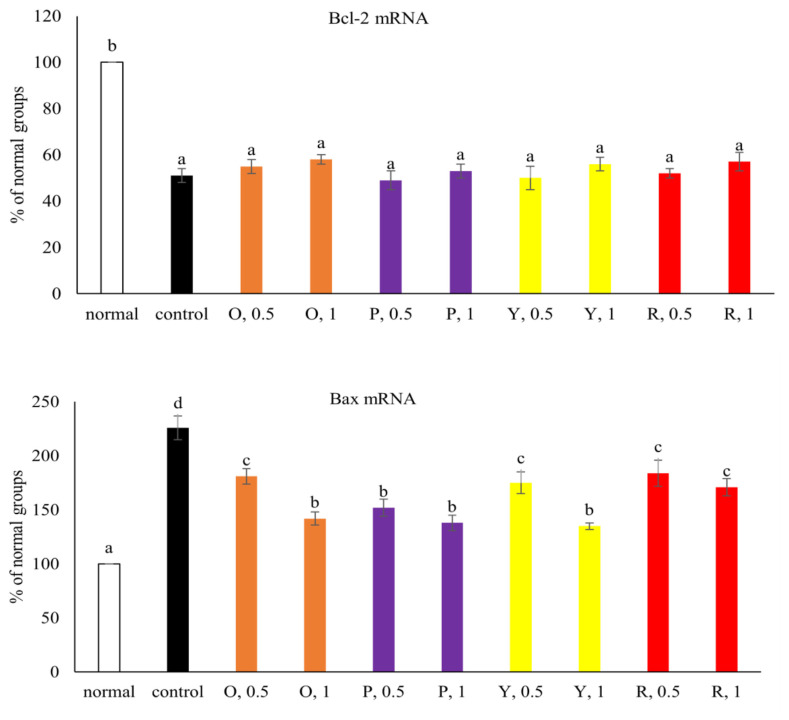
Effects of aqueous extracts prepared from orange (O), purple (P), yellow (Y) and red (R) peppers upon mRNA expression of Bcl-2 and Bax. NGF-differentiated PC12 cells were pre-treated with aqueous extracts at 0.5 and 1 mg for 48 h at 37 °C, and followed by 33 mM glucose treatment for 24 h. Normal groups were NGF-differentiated PC12 cells treated with 5.5 mM glucose and without aqueous extracts. Control groups were NGF-differentiated PC12 cells treated with 33 mM glucose and without aqueous extracts. Data were expressed as mean ± SD (n = 7). ^a–d^Values among bars without a common letter differ, P < 0.05.

High glucose decreased the survival of NGF-differentiated PC12 cells (Fig. 4, *P* < 0.05). Pretreatments of pepper aqueous extracts at 0.5 and 1 mg increased the viability of NGF-differentiated PC12 cells (*P* < 0.05). At equal concentration, orange and purple peppers showed greater effects in increasing cell survival than yellow and red peppers (*P* < 0.05). Pepper aqueous extracts at both 0.5 and 1 mg lowered LDH activity (*P* < 0.05), in which orange and purple peppers at 1 mg showed greater effects than 0.5 mg treatments (*P* < 0.05). As shown in Fig. 5, high glucose lowered Bcl-2 mRNA expression and increased Bax mRNA expression (*P* < 0.05). Pepper aqueous extracts failed to affect Bcl-2 mRNA expression (*P* > 0.05), but at both 0.5 and 1 mg decreased Bax mRNA expression (*P* < 0.05). High glucose decreased MMP and Na^+^-K^+^ ATPase activity (Table 2, *P* < 0.05). Pepper aqueous extracts at both 0.5 and 1 mg increased MMP and Na^+^-K^+^ ATPase activity (*P* < 0.05). Purple pepper at 1 mg had the greatest effects in elevating both MMP and Na^+^-K^+^ ATPase activity (*P* < 0.05).

### 3.5. DNA fragmentation and Ca^2^^+^ release

**Fig. 6:**
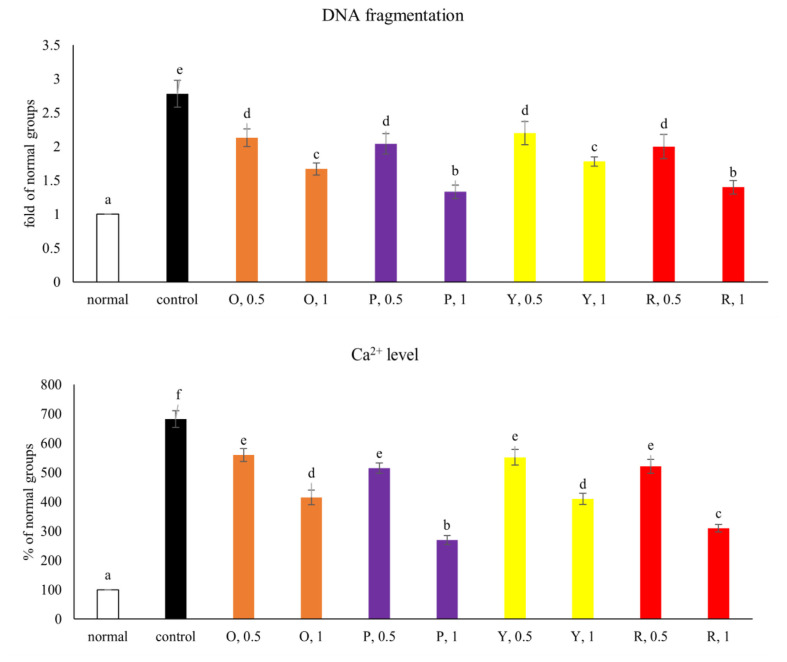
Effects of aqueous extracts prepared from orange (O), purple (P), yellow (Y) and red (R) peppers upon DNA fragmentation and intracellular Ca^2+^ level. NGF-differentiated PC12 cells were pre-treated with aqueous extracts at 0.5 and 1 mg for 48 h at 37 °C, and followed by 33 mM glucose treatment for 24 h. Normal groups were NGF-differentiated PC12 cells treated with 5.5 mM glucose and without aqueous extracts. Control groups were NGF-differentiated PC12 cells treated with 33 mM glucose and without aqueous extracts. Data were expressed as mean ± SD (n = 7). ^a–f^Values among bars without a common letter differ, P < 0.05.

As shown in Fig. 6, high glucose increased DNA fragmentation and intracellular Ca^2+^ level in NGF-differentiated PC12 cells (*P* < 0.05). Pepper aqueous extracts at both 0.5 and 1 mg decreased DNA fragmentation (*P* < 0.05), in which purple and red pepper at 1 mg had lower DNA fragmentation than other peppers (*P* < 0.05). Pepper aqueous extracts also at both 0.5 and 1 mg lowered intracellular Ca^2+^ level (*P* < 0.05), in which purple pepper at 1 mg had the lowest intracellular Ca^2+^ level (*P* < 0.05).

### 3.6. Anti-oxidative and anti-inflammatory effects

As shown in Table 3, high glucose increased ROS level, reduced GSH content, lowered GPX and GR activities in NGF-differentiated PC12 cells (*P* < 0.05).

Pre-treatments of pepper aqueous extracts at 0.5 and 1 mg decreased ROS level, increased GSH content, GPX and GR activities (*P* < 0.05). High glucose increased TNF-alpha, IL-1beta and IL-6 levels in NGF-differentiated PC12 cells (Table 4, *P* < 0.05).

Pre-treatments of pepper aqueous extracts at 0.5 and 1 mg decreased the level of these cytokines (*P* < 0.05).

## 4. Discussion

These colored peppers are consumed as vegetables in Taiwan and other countries. Our present study found that these colored peppers contained vitamin C, phenolic acids, flavonoids, anthocyanins and triterpenoids. In addition, the aqueous extracts prepared from these peppers displayed marked *in vitro* anti-oxidative, anti-α-amylase, anti-α-glucosidase, anti-lipase and anti-AchE activities. Furthermore, the data of cell line study revealed that the aqueous extracts from these peppers could protect NGF-differentiated PC12 cells against high glucose induced apoptotic, oxidative and inflammatory damage. These novel findings suggest that four colored peppers could offer multiple nutritional advantages for consumers.

Vitamin C is a required nutrient for human, and involved in many crucial physiological functions like collagen synthesis, iron absorption, radical elimination and pleiotropic benefits in critical illness [24,25]. Since these colored peppers, especially red pepper, contained substantial level of vitamin C, these peppers could be considered as natural sources of vitamin C. It has been documented that vitamin C and phytochemicals possess anti-oxidative functions [1,3,26]. Thus, the observed DPPH scavenging effects, iron-chelating effects and reducing power, in these colored peppers may be owing to the presence of vitamin C and four kinds of phytochemicals. Via these anti-oxidative actions, the intake of aqueous extracts from these peppers might be able to prevent and attenuate some oxidative associated diseases such as diabetes and AD [27,28].

Decreased activity of α-amylase and α-glucosidase could delay the degradation of CHO such as starch and oligosaccharides to glucose, which consequently reduce postprandial hyperglycemia [29,30]. In our present study, aqueous extracts from colored peppers markedly limited the activity of both α-amylase and α-glucosidase. These results suggest that these peppers might improve glycemic control via declining the activity of these two enzymes. Decreased pancreatic lipase activity could diminish intestinal lipid catabolism, and decrease available lipids for the development of hyperlipidemia and obesity [31]. It is known that increased AchE activity in brain resulted in lower available acetylcholine for brain bio-functions, which promotes AD pathogenic progression [32]. Our present study found that pepper aqueous extracts displayed *in vitro* anti-lipase and anti-AchE activities. These data implied that these colored peppers via restricting lipase and AchE activities might be able to attenuate hyperlipidemia, steatosis and AD. It is documented that some phenolic acids, flavonoids, anthocyanins and triterpenoids could improve hyperglycemia, obesity and AD through limiting the activity of α-amylase, α-glucosidase, lipase and AchE [33–35]. Since colored peppers were rich in those phytochemicals, the observed inhibition from colored peppers upon four enzymes could be ascribed to the actions of phytochemicals.

High glucose induced oxidative, inflammatory and apoptotic damage, led to loss of Schwann cells, caused nerve deficits and promoted DN progression [36,37]. In our present study, high glucose decreased Bcl-2 mRNA expression and increased Bax mRNA expression, which partially explained the observed apoptosis of NGF-differentiated PC12 cells. However, the pre-treatments of pepper extracts markedly diminished the mRNA expression of Bax, an apoptotic factor, which in turn attenuated high glucose induced apoptotic stress and benefited the survival of NGF-differentiated PC12 cells. Obviously, those colored pepper extracts could exert protective actions at molecular level through mediating apoptotic elements. Furthermore, high glucose evoked oxidative and inflammatory stress in NGF-differentiated PC12 cells, which were evidenced by greater production of ROS, TNF-alpha, IL-1beta and IL-6, lower GSH content and decreased GPX and GR activities. Consequently, cellular and mitochondrial membranes were disturbed, LDH was leaked, MMP and Na^+^-K^+^ ATPase activity were diminished, DNAs were fragmented and Ca^2+^ was released. Finally, cell death was observed. However, our data revealed that aqueous extracts from colored peppers effectively ameliorated high glucose induced oxidative and inflammatory damage, stabilized DNAs, maintained cellular and mitochondrial membranes integrity, favored Ca^2+^ homeostasis and enhanced cell survival.

These results indicated that these colored peppers were potent anti-DN agents.

Vitamin C based on its anti-oxidative and antiinflammatory properties has been used to treat DN related pain and diabetic cardiac autonomic neuropathy [38,39]. Since vitamin C content in these colored peppers was remarked, the observed antioxidative and anti-inflammatory protection from these peppers in high glucose treated NGF-differentiated PC12 cells could be partially explained. In addition, phytochemicals such as triterpenes, resveratrol and flavonoids have been used for DN therapy [40,41]. Since four examined colored peppers were rich in phytochemicals, it is highly possible that these phytochemicals contributed to the observed neuro-protective effects of colored peppers. These results suggested that active component compounds such as vitamin C and phytochemicals in pepper aqueous extracts could penetrate NGF-differentiated PC12 cells, and exert anti-oxidative, anti-inflammatory and membrane-protective actions. Thus, the greater cell survival observed in pepper treated NGF-differentiated PC12 cells could be ascribed to the actions of vitamin C, phytochemicals or the interactions between vitamin C and phytochemicals.

It is reported that high glucose induced DNA fragmentation, and led to cell rupture and death [42]. Disturbance of neuronal Ca^2+^ homeostasis caused synaptic deficits and neuronal excitotoxicity, which facilitated malfunction and death of neuronal cells [43,44].

Thus, both DNA stability and Ca^2+^ homeostasis are crucial factors for DN prevention and alleviation. Our data indicated that aqueous extracts prepared from peppers, especially purple pepper, effectively retarded high glucose induced DNA fragmentation and massive.

Ca^2+^ release, which in turn benefited the survival of NGF-differentiated PC12 cells. These findings once again implied that active component compounds of pepper aqueous extracts could penetrate NGF-differentiated PC12 cells and perform their anti-apoptotic actions through stabilizing DNAs and maintaining Ca^2+^ homeostasis.

## 5. Conclusions

Four colored peppers contained substantial levels of vitamin C and four kinds of phytochemicals. Aqueous extracts prepared from these colored peppers displayed markedly *in vitro* anti-oxidative, anti-α-amylase, anti-α-glucosidase, anti-lipase and anti-AchE activities. In addition, these aqueous extracts exhibited anti-oxidative, anti-inflammatory and anti-apoptotic protection for NGF-differentiated PC12 cells against high glucose. Furthermore, these aqueous extracts reversed high glucose induced Bax mRNA expression, DNA fragmentation and Ca^2+^ dyshomeostasis in NGF-differentiated PC12 cells. These findings suggest that these colored peppers might provide multiple bio-functions to prevent or alleviate diabetic complications such as DN.

## Figures and Tables

**Table 1:** Content of vitamin C (mg/100 g fresh weight), total phenolic acids (mg gallic acid equivalents/100 g DW), total flavonoids (mg quercetin equivalents/100 g DW), total anthocyanins (mg cyanidin-3-glucoside equivalents/100 g DW) and total triterpenoids (mg ursolic acid equivalents/ 100 g DW) in aqueous extracts prepared from orange (O), purple 5 (P), yellow (Y) and red (R) peppers. Data were expressed as mean ± SD (n = 7).

	O	P	Y	R
Vitamin C	82±5^b^	60±3^a^	76±4^b^	96±7^c^
Total phenolic acids	1005±62^a^	1081±104^a^	926±59^a^	919±78^a^
Total flavonoids	1391±97^b^	903±112^a^	1275±138^b^	1416±85^b^
Total anthocyanins	1672±134^a^	1960±93^b^	1453±156^a^	1581±170^a^
Total triterpenoids	1795±87^b^	2185±148^c^	1526±121^a^	1387±99^a^

a–c Values among rows without a common letter differ, *P* < 0.05.

**Table 2:** Effects of aqueous extracts prepared from orange (O), purple (P), yellow (Y) and red (R) peppers upon MMP and Na^+^-K^+^ ATPase activity. NGF-differentiated PC12 cells were pre-treated with aqueous extracts at 0.5 and 1 mg for 48 h at 37 °C, and followed by 33 mM glucose treatment for 24 h. Normal groups were NGF-differentiated PC12 cells treated with 5.5 mM glucose and without aqueous extracts. Control groups were NGF-differentiated PC12 cells treated with 33 mM glucose and without aqueous extracts. Data were expressed as mean ± SD (n = 7).

	concentration, mg	MMP	Na^+^-K^+^ ATPase
Normal		100^e^	100^f^
Control		25±2^a^	32±3^a^
O	0.5	42±4^b^	48±2^b^
	1	58±3^c^	64±3^d^
P	0.5	52±5^b^	56±4^c^
	1	71±2^d^	75±6^e^
Y	0.5	49±4^b^	50±3^b^
	1	68±3^d^	63±2^d^
R	0.5	40±5^b^	54±1^c^
	1	56±3^c^	73±5^e^

a–f Values in a column without a common letter differ, *P* < 0.05.

**Table 3:** Effects of aqueous extracts prepared from orange (O), purple (P), yellow (Y) and red (R) peppers upon ROS level (RFU/mg protein), GSH content (nmol/mg protein), GPX and GR activities (U/mg protein). NGF-differentiated PC12 cells were pre-treated with aqueous extracts at 0.5 and 1 mg for 48 h at 37 °C, and followed by 33 mM glucose treatment for 24 h. Normal groups were NGF-differentiated PC12 cells treated with 5.5 mM glucose and without aqueous extracts. Control groups were NGF-differentiated PC12 cells treated with 33 mM glucose and without aqueous extracts. Data were expressed as mean ± SD (n = 7).

	concentration, mg	ROS	GSH	GPX	GR
Normal		0.11±0.04^a^	92±3^d^	74.2±1.7^e^	63.4±2.2^e^
Control		2.88±0.25^e^	30±4^a^	36.1±2.0^a^	32.5±1.9^a^
O	0.5	2.21±0.15^d^	45±5^b^	41.5±1.8^b^	37.0±1.3^b^
	1	1.74±0.1^c^	59±3^c^	49.6±1.1^c^	45.2±2.0^c^
P	0.5	2.13±0.19^d^	48±2^b^	42.3±0.9^b^	40.5±1.6^b^
	1	1.25±0.08^b^	63±4^c^	50.9±2.1^c^	53.7±1.2^d^
Y	0.5	2.27±0.21^d^	43±1^b^	48.8±1.5^c^	39.9±2.1^b^
	1	1.69±0.14^c^	67±3^c^	59.5±1.2^d^	50.4±0.7^d^
R	0.5	2.18±0.09^d^	47±5^b^	43.0±1.3^b^	41.2±1.5^b^
	1	1.84±0.12^c^	65±2^c^	58.4±1.8^d^	54.5±1.8^d^

a–e Values in a column without a common letter differ, *P* < 0.05.

**Table 4:** Effects of aqueous extracts prepared from orange (O), purple (P), yellow (Y) and red (R) peppers upon level (pg/mg protein) of TNF-alpha, IL-1beta and IL-6. NGF-differentiated PC12 cells were pre-treated with aqueous extracts at 0.5 and 1 mg for 48 h at 37 °C, and followed by 33 mM glucose treatment for 24 h. Normal groups were NGF-differentiated PC12 cells treated with 5.5 mM glucose and without aqueous extracts. Control groups were NGF-differentiated PC12 cells treated with 33 mM glucose and without aqueous extracts. Data were expressed as mean ± SD (n = 7).

	concentration, mg	TNF-alpha	IL-1beta	IL-6
Normal		10±3^a^	12±5^a^	13±2^a^
Control		189±17^e^	159±11^e^	174±15^d^
O	0.5	135±9^d^	125±12^d^	135±10^c^
	1	84±7^b^	92±6^c^	97±8^b^
P	0.5	112±11^c^	116±7^d^	129±13^c^
	1	73±8^b^	68±9^b^	94±5^b^
Y	0.5	148±13^d^	123±10^d^	131±9^c^
	1	108±10^c^	75±8^b^	100±12^b^
R	0.5	139±7^d^	120±5^d^	140±11^c^
	1	89±5^b^	93±4^c^	103±6^b^

a–e Values in a 8 column without a common letter differ, *P* < 0.05.
